# Nomogram model for predicting secondary infection in critically ill patients with heatstroke: A pilot study from China

**DOI:** 10.1371/journal.pone.0316254

**Published:** 2024-12-26

**Authors:** Guodong Lin, Hailun Peng, Bingling Yin, Chongxiao Xu, Yueli Zhao, Anwei Liu, Haiyang Guo, Zhiguo Pan

**Affiliations:** 1 The First Clinical Medical College, Southern Medical University, Guangzhou, Guangdong, China; 2 Department of Critical Care Medicine, General Hospital of Southern Theatre Command of PLA, Guangzhou, Guangdong, China; 3 Department of Critical Care Medicine, Longgang Central Hospital of Shenzheng, Shenzheng, Guangdong, China; 4 Department of Graduate School, Guangzhou University of Chinese Medicine, Guangzhou, Guangdong, China; 5 Department of Emergency Medicine, Weifang People’s Hospital, Weifang, Shandong, China; 6 Department of Emergency Medicine, General Hospital of Southern Theatre Command of PLA, Guangzhou, Guangdong, China; Swansea University, UNITED KINGDOM OF GREAT BRITAIN AND NORTHERN IRELAND

## Abstract

**Objective:**

In this retrospective analysis, we explored the clinical characteristics and risk factors of secondary infections in patients with severe heatstroke with the aim to gain epidemiological insights and identify risk factors for secondary infections.

**Method:**

The study included 129 patients with severe heatstroke admitted to the General Hospital of the Southern Theater Command of the PLA between January 1, 2011, and December 31, 2021. Patients were divided into an infection group (n = 24) and a non-infection group (n = 105) based on infection occurrence within 48 h of intensive care unit (ICU) admission. Clinical indicators, infection indicators, and clinical outcomes within 24 h of ICU admission were collected and compared between the groups. Independent risk factors for infection in patients with severe heatstroke were analyzed using univariate and multivariate analyses. A nomogram model was constructed, evaluated, and validated.

**Result:**

Among the 129 patients with heatstroke, 24 developed secondary infections. Infections occurred between days 3 and 10 post-ICU admission, primarily affecting the lungs. Multivariate analysis identified vasopressor use, serum creatinine level, and gastrointestinal dysfunction at admission as independent risk factors, while elevated lymphocyte count (odds ratio [OR] = 0.167; 95% confidence interval [CI] 0.049–0.572; P = 0.004) was protective against severe heatstroke. Infected patients required longer durations of mechanical ventilation (OR = 2.764; 95% CI, 1.735–4.405; P = 0.044) and total hospital stay than those in the non-infection group. The nomogram model demonstrated clinical feasibility.

**Conclusion:**

Increased lymphocyte count is an independent protective factor against infections in patients with severe heatstroke. Vasopressor use, gastrointestinal dysfunction, and elevated serum creatinine levels are independent risk factors. These indicators can aid clinicians in assessing infection risk in patients with severe heatstroke.

## Introduction

Owing to the increased production of greenhouse gases from human activities contributing to climate change, the frequency of severe heat waves worldwide has increased significantly [1]. In the United States, the number of heat waves tripled to six per year between 1960 and 2010 [2]. Heatstroke accounts for the highest proportion of morbidity and mortality among heat-related illnesses during heatwaves [3]. The continuous progression to severe heatstroke, accompanied by single or multiple organ dysfunction or failure, can result in high mortality rates [4]. The central nervous system, coagulation system, kidneys, liver, and skeletal muscle are particularly susceptible to damage after heatstroke [5]. Additionally, heatstroke can significantly affect the immune function of patients [6, 7].

The early stage of heatstroke typically manifests as a non-infectious systemic inflammatory response. Once a heatstroke occurs, the body’s inflammatory immune state is disturbed, which may increase the chance of infection. However, the clinical characteristics of patients with infections after heatstroke have not been well documented. Infections may worsen the condition of critically ill patients and may increase mortality once secondary infections occur.

This retrospective study sought to understand the clinical characteristics and risk of secondary infections in patients with severe heatstroke. To this end, data for patients with severe heatstroke were evaluated, and a nomogram was constructed to guide clinicians in assessing patients’ risk of secondary infections.

## Methods

### Study design and setting

The study participants were patients admitted to the General Hospital of the Southern Theater Command of the PLA Hospital between January 1, 2011, and December 31, 2021, due to heatstroke. The data were obtained on November 20, 2022. All data were stripped of personal identifying information. The institutional ethics committee waived the need for informed consent owing to the retrospective design of the study. This study adhered to the medical ethics standards and was approved by the Ethics Committee of the Southern Theater General Hospital (approval number: NZLLKZ2022099).

### Patient selection and grouping

Patients diagnosed with heatstroke, who received treatment in the intensive care unit (ICU) for more than 48 h and had complete case data were included in this study. Heatstroke diagnoses adhered to the expert consensus on the diagnosis and treatment of heatstroke in China [8]. To distinguish between infection and colonization, the secondary infection criteria were: (1) worsening of existing symptoms or the onset of new symptoms such as chills and fever; (2) elevated white blood cell count (> 15.00 × 10^9/L), elevated procalcitonin levels, or imaging that supports the diagnosis of infection in the corresponding area [9]. The following exclusion criteria were applied: (1) infections before ICU admission or infections within 24–48 h after ICU admission; (2) ICU treatment before admission to our hospital; (3) cardiac arrest due to the primary disease and complications after cardiopulmonary resuscitation; and (4) malignant tumors, autoimmune diseases, or mental disorders.

### Data collection and variables

Data collection primarily included recording baseline patient information such as age and sex; vital signs at admission, including body temperature (T), heart rate, mean arterial pressure, and blood glucose; mechanical ventilation use; vasopressor use; indicators of organ damage; deep vein catheterization duration (days), urinary catheter indwelling duration (days), broad-spectrum antibiotic use, antibiotic duration (days), hemofiltration duration, time from admission to secondary infection (days), infection site, pathogen, septic shock incidence, gastrointestinal dysfunction, diffuse intravascular coagulation (DIC), length of mechanical ventilation, length of ICU stay, total length of hospital stay, acute gastrointestinal functional impairment classification, and Glasgow Coma Scale (GCS), International Society of Thrombosis and Hemostasis (ISTH) DIC, Systemic Inflammatory Response Syndrome (SIRS), Sequential Organ Failure Assessment (SOFA), and Acute Physiology and Chronic Health Evaluation II (APACHE II) scores.

### Statistical analysis

SPSS Statistics 26.0 and R4.2.1 software were used for statistical analysis. Continuous variables that conformed to a normal distribution are presented as mean ± standard deviation (SD), while those that did not conform to a normal distribution are expressed as median and quartiles (25%, 75%). Categorical variables are presented as numbers (percentages). An independent Sample *t*-test was used to compare two independent samples with a normal distribution, whereas the nonparametric Mann–Whitney U-test was used to compare two independent samples that were not normally distributed. The Kruskal–Wallis rank sum test was used to compare multiple independent samples that were not normally distributed, and the chi-squared test or Fisher’s exact probability method was used to test contingency tables. Significant indices were obtained through univariate logistic regression; these variables were subsequently included in multivariate logistic regression analysis. The Forward likelihood ratio method was employed to screen variables and evaluate independent predictors of secondary infection in patients with heatstroke. Additionally, receiver operating characteristic (ROC) curve analysis was used to determine the critical value of the positive indices for predicting secondary infection risk. Univariate and multivariate logistic regression analyses were conducted to analyze the effects of secondary infections on patient outcomes. The nomogram was developed using the R software, and the model’s credibility and clinical application value were evaluated by plotting calibration curves and decision curves/clinical impact curves. Statistical significance was set at P < 0.05.

## Results

### Grouping according to secondary infection occurrence

**Fig 1:**
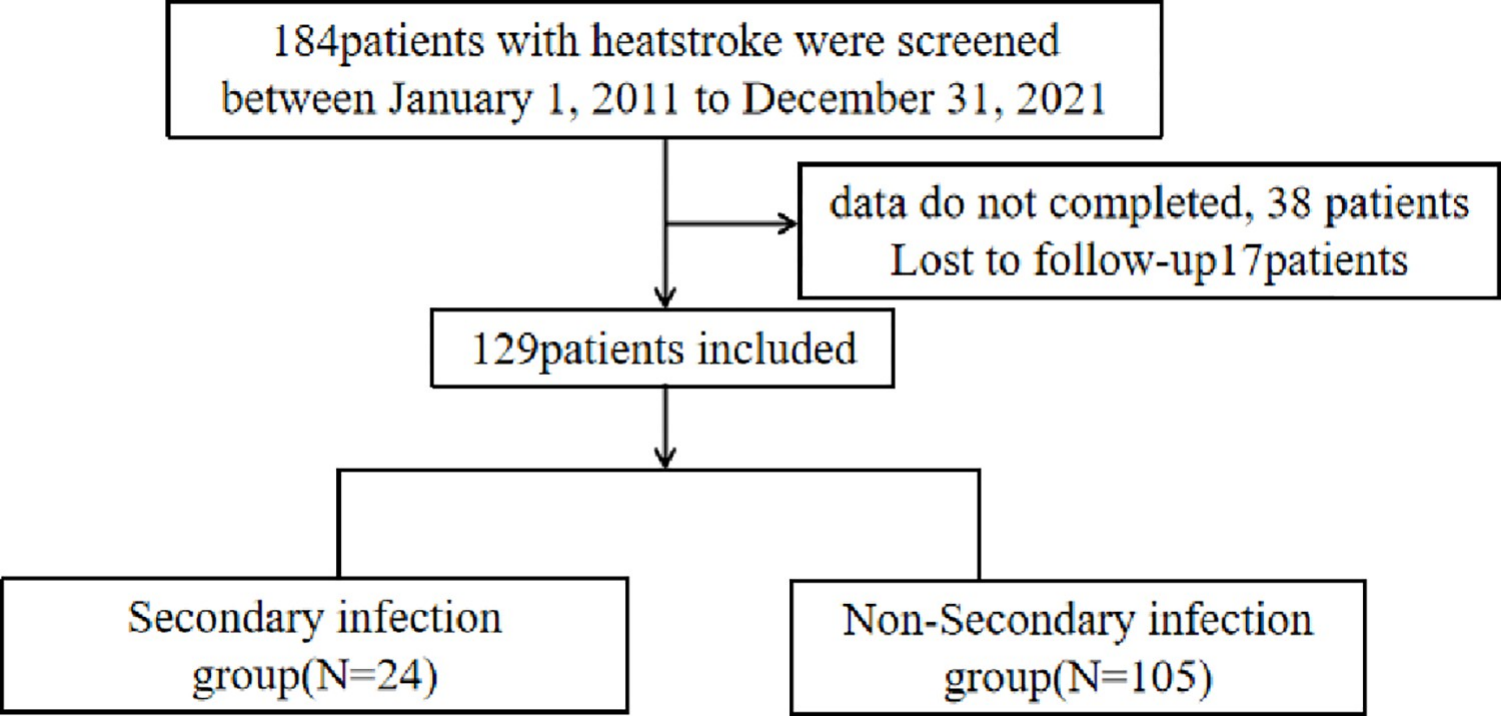
Flowchart of patients with severe heatstroke grouped according to whether they had secondary infections.

A total of 184 patients with severe heatstroke were treated in the ICU of the Southern Theater General Hospital between January 1, 2011, and December 31, 2021. Based on the inclusion and exclusion criteria, 38 patients with incomplete case data and short ICU stay were excluded. Seventeen patients died during follow-up. Patients with severe heatstroke were divided into two groups based on whether they had an infection after entering the ICU: a secondary infection group and a non-infection group (Fig 1). There were 24 cases (18.6%) of secondary infection and 105 cases (81.4%) without infection.

### Comparison of clinical data between ICU patients with heatstroke and with or without infections

Compared with the ICU group with non-secondary infections following severe heatstroke, admission temperature, heart rate, mechanical ventilation use, vasopressor use (primarily norepinephrine), serum total bilirubin, glutamic oxalic transaminase, creatinine, creatine kinase, prothrombin time, international normalized ratio, activated partial thromboplastin time, procalcitonin, SOFA score, APACHE II score, ISTH score, DIC incidence, multiple organ dysfunction syndrome (MODS) incidence, gastrointestinal dysfunction incidence, intestinal decontamination proportion, central venous catheter use proportion, central venous catheterization time, urinary tube use, urinary catheterization time, broad-spectrum antibiotic use, and duration of antibiotic use were all higher in the secondary infection group, while the platelet count and GCS score were lower than in the non-infection group (Table 1). Hence, the secondary infection group had more severe damage to pertinent organ systems (respiratory, circulatory, liver, kidney, coagulation, rhabdoid muscle, and central nervous system) than the non-infection group. Additionally, they had higher scores regarding inflammation, infection, and disease severity and received more antibiotics and invasive procedures. However, age, blood glucose level, white blood cell count, neutrophil count, and C-reactive protein level did not differ significantly between the groups.

**Table 1:** Comparison of baseline characteristics between ICU patients with severe heatstroke in the non-secondary infection and infection groups.

Variable	Non-infection group, No. (%) (n = 105)	Infection group, No. (%) (n = 24)	P values
Age (Y)	23 (19–26)	24 (18–25)	0.959
Vital sign			
Temperature (°C)	37.2 (36.5–37.8)	37.7 ± 1.2	0.010
Heart rate (bpm)	84 (70–90)	115 ± 23	< 0.001
Mean arterial pressure (mmHg)	83 ± 10	88 (76–95)	0.228
Mechanical ventilation n (%)	5 (4.8%)	10 (41.7%)	< 0.001
Vasopressors n (%)	2 (1.9%)	7 (29.2%)	< 0.001
Organ damage variable			
TBIL (μmol/L)	19.9 (10.6–25.3)	39.9 (12.5–64.8)	0.014
AST (U/L)	351 (28–139)	1611 (110–2095)	< 0.001
SCR (μmol/L)	113 (82–132)	202 ± 78	< 0.001
CK (U/L)	3038 (279–2525)	4112 (1055–7010)	0.001
PT (sec)	18.5 (14.9–18.9)	29.9 (20.6–38.2)	< 0.001
APTT (sec)	50.0 (35.1–43.9)	101.5 (47.2–147.1)	< 0.001
PLT (10^9^/L)	152 ± 63	75 ± 56	< 0.001
WBC (10^9^/L)	12.6 (9.5–15.1)	12.4 (8.5–15.7)	0.509
NEU (10^9^/L)	10.6 (7.3–13.2)	10.4 (6.6–12.9)	0.672
LYM (10^9^/L)	1.2 (0.6–1.7)	1.1 (0.4–1.4)	0.141
PCT (ng/mL)	2.7 (0.2–2.9)	4.7 (1.6–6.5)	0.001
GCS	14 (9–15)	8 (4–15)	< 0.001
DIC n (%)	15 (14.3%)	17 (70.8%)	< 0.001
MODS n (%)	19 (18.1%)	21 (87.5%)	< 0.001
Septic shock n (%)	1 (1.0%)	13 (54.2%)	< 0.001
Infection-related variable			
GD n (%)	10 (9.5%)	20 (83.3%)	< 0.001
Intestinal decontamination n (%)	20 (19.0%)	23 (95.8%)	< 0.001
Central venous catheter n (%)	58 (55.2%)	24 (100%)	< 0.001
Urinary catheter n (%)	68 (64.8%)	24 (100%)	0.001
Antibiotic use n (%)	43 (41.0%)	24 (100%)	< 0.001
Central venous catheter time (day)	2 (0–4)	9 (5–13)	< 0.001
Urinary catheter time (day)	1 (0–4)	11 (6–20)	< 0.001
Time of antibiotic use (day)	0 (0–4)	10 (7–19)	< 0.001
Illness severity			
SOFA	3 (1–4)	10 ± 6	< 0.001
APACHEII	5 (2–6)	14 ± 8	< 0.001
Outcome			
MV LOS, day	0 (0–0)	6 (1–9)	< 0.001
Mortality n (%)	2 (1.9%)	12 (50.0%)	< 0.001
ICU LOS, day	7 (3–7)	16 (8–20)	< 0.001
HOS LOS, day	17 (4–19)	50 (9–70)	0.002

Abbreviations: TBIL: total bilirubin, AST: aspartate aminotransferase, SCR: serum creatinine, CK: creatine kinase, PT: prothrombin time, APTT: activated partial thromboplastin time, PLT: platelet, WBC: white blood cells, NEU: neutrophil count, LYM: lymphocyte count, PCT: procalcitonin, GCS: Glasgow Coma Scale, SOFA: Sequential Organ Failure Assessment, APACHEII: Acute Physiology and Chronic Health Evaluation II, DIC: Disseminated Intravascular Coagulation, MODS: multiple organ dysfunction syndrome, GD: gastrointestinal dysfunction, MV LOS: length of mechanical ventilation, ICU LOS: length of ICU stay, HOS LOS: total length of hospital stay.

In terms of clinical outcomes, the incidence of septic shock, hospital death, median length of mechanical ventilation, median length of ICU stay, and median length of hospital stay were higher in the infection group than in the non-infection group.

### Characteristics of patients with severe heatstroke and infections

**Fig 2:**
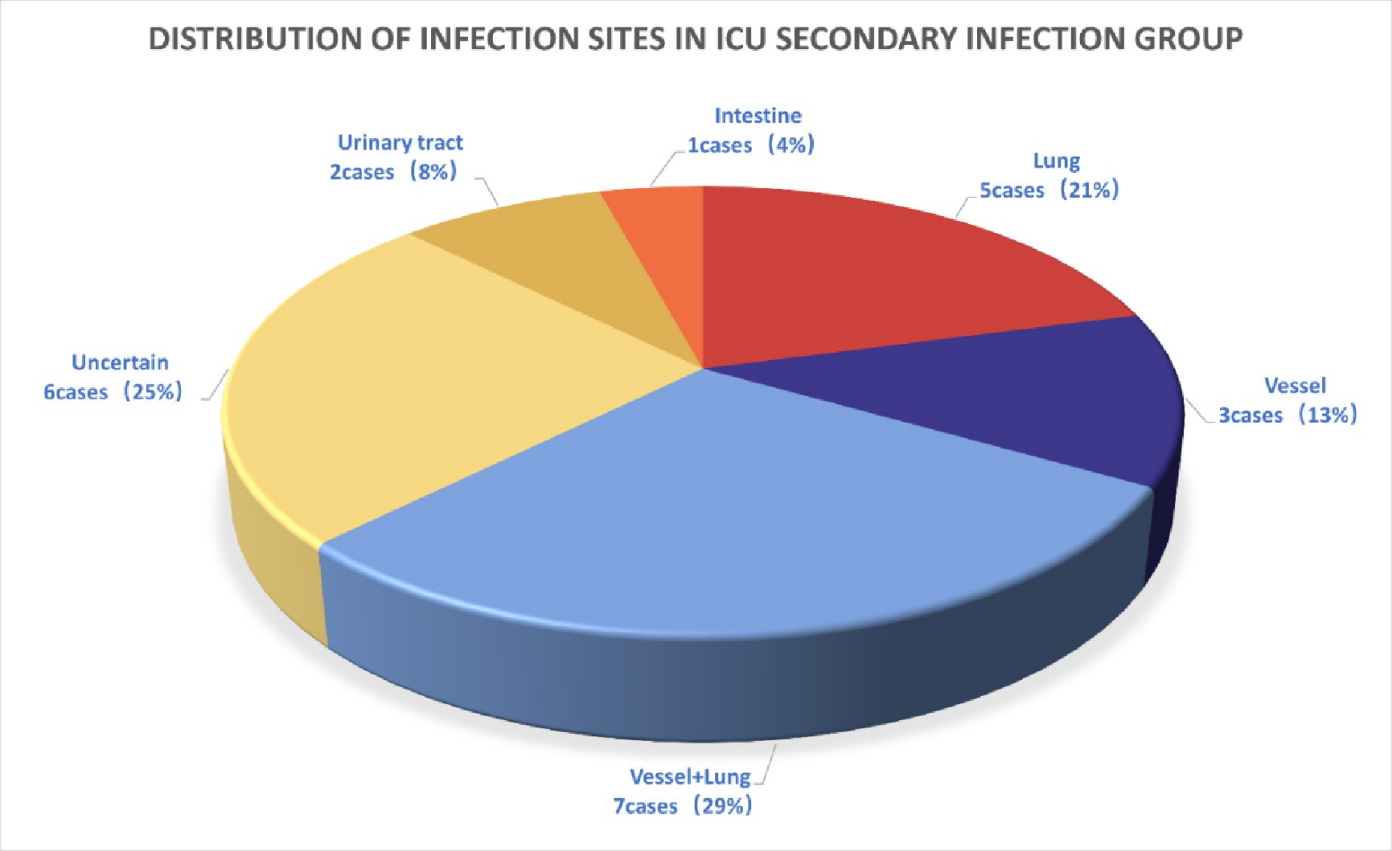
Distribution of infected sites in the infection group of ICU patients with heatstroke.

The infection site, occurrence time of secondary infection, and pathogen distribution for the 24 patients with heatstroke and infections were statistically analyzed. The main infection sites in the ICU heatstroke infection group were the lungs and blood vessels (Fig 2). There were seven cases of blood infection complicated by pulmonary infection (29.0%) and five cases of pulmonary infection alone (21%). Cumulatively, there were 12 cases of pulmonary infection (50%), 3 of vascular infection (13%), 2 of urinary tract infection (8%), 1 of intestinal infection (4%), and 6 of unknown infection (25%).

**Fig 3:**
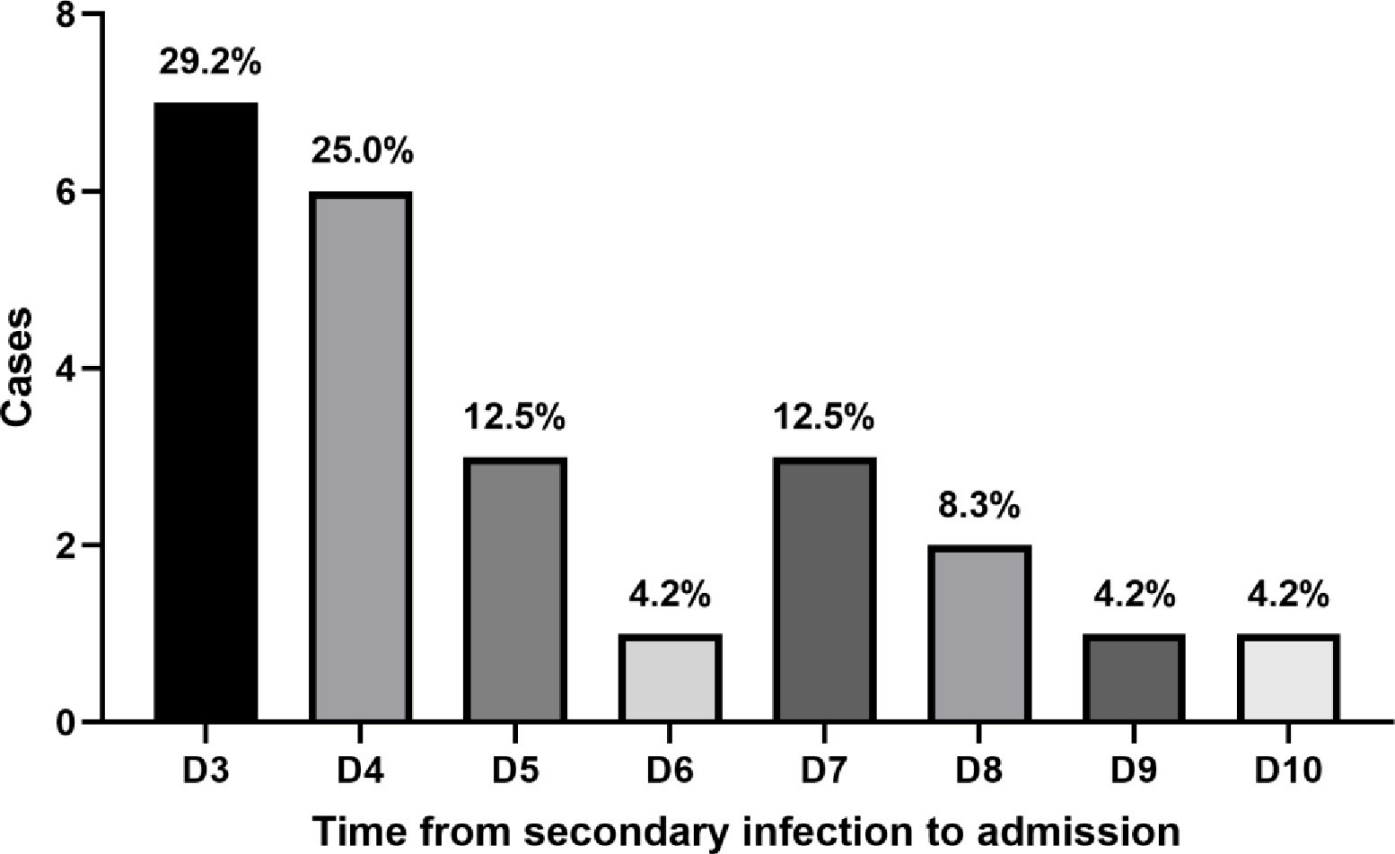
Distribution of secondary infection cases and time of infection in ICU heatstroke.

Infections typically occurred 3–10 days after ICU admission (Fig 3). Among the selected cases, the occurrence of infections peaked between days 3 and 5 after ICU admission, with seven cases on day 3 (29.2%), six on day 4 (25.0%), and three on day 5 (12.5%). Altogether, 22 patients (91.7%) developed infection within 8 days of ICU admission.

**Fig 4:**
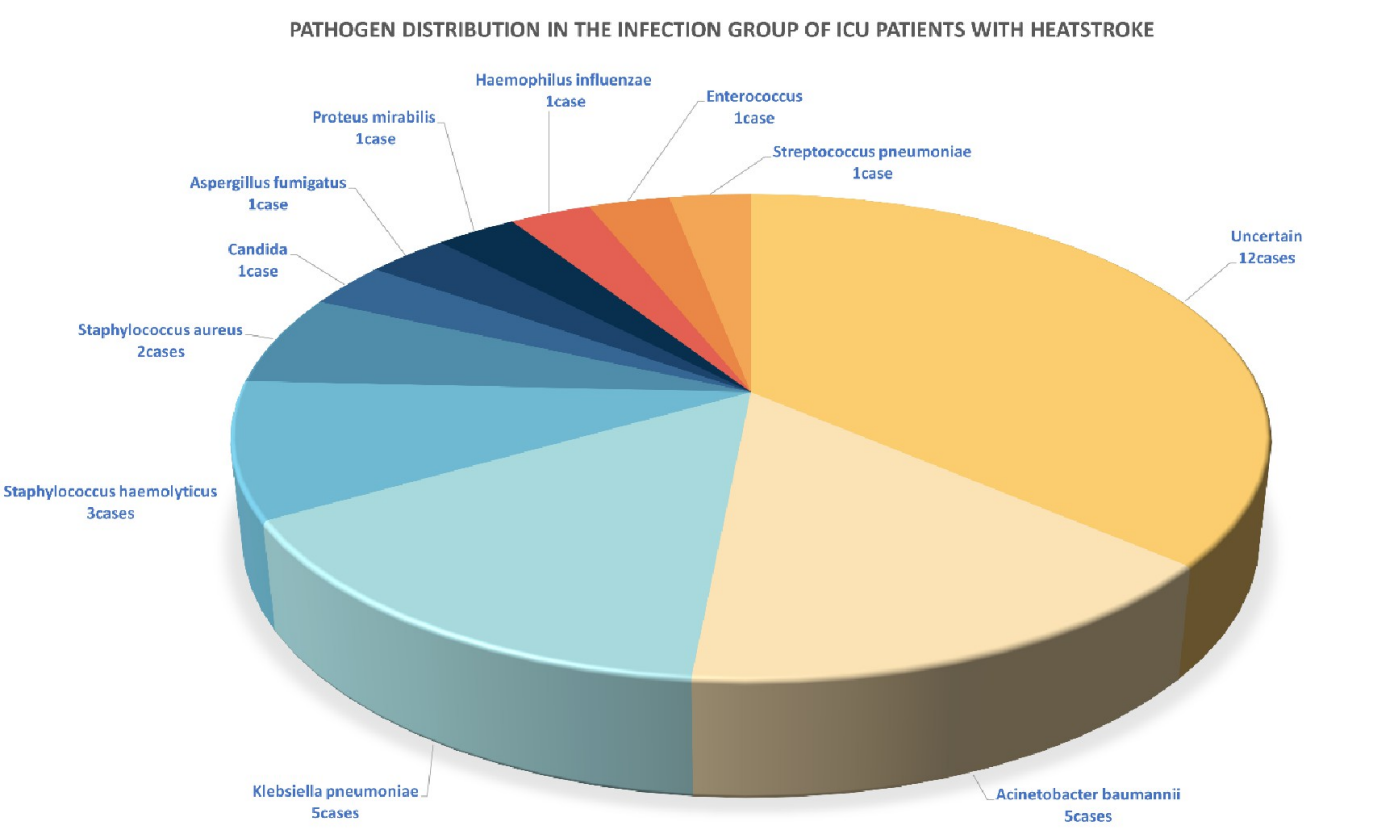
Pathogen distribution in the infection group of ICU patients with heatstroke.

The distribution of pathogens in the heatstroke infection group (Fig 4) included 12 cases of pathogen infection in the secondary infection group, all of which were multidrug-resistant bacteria. Carbapenem-resistant *Acinetobacter baumannii* and *Klebsiella pneumoniae* were the most common pathogens. Five cases (20.8%) were single or co-infected with *Acinetobacter baumannii*, and four (16.7%) were single or co-infected with *Klebsiella pneumoniae*. There were two cases of fungal infection, accounting for 8.3% of the cases.

### Univariate and multivariate analyses of risk factors for infection in patients with heatstroke

The statistically significant variables, including body temperature, heart rate, Vasopressor use, total bilirubin, serum creatinine levels, platelet count, gastrointestinal dysfunction, GCS score, mechanical ventilation use, and SOFA score, were included in the multivariate analysis (Table 2). Vasopressor use (odds ratio [OR] = 45.467; 95% confidence interval [CI], 1.460–1416.275; P = 0.030), serum creatinine levels (OR = 1.026; 95% CI, 1.005–1.048; P = 0.017), and gastrointestinal dysfunction (OR = 14.338; 95% CI, 0.873–1.795; P = 0.045) were independent risk factors for predicting secondary infection in patients with heatstroke, whereas the lymphocyte count at admission (OR = 0.167; 95% CI 0.049–0.572; P = 0.004) was an independent protective factor for secondary infection in patients with heatstroke. Meanwhile, age, mechanical ventilation use, body temperature, heart rate, total bilirubin, glutamic oxaloacetic transaminase, creatine kinase, prothrombin time, platelet count, white blood cell count, procalcitonin level, C-reactive protein level, and SOFA scores did not differ significantly.

**Table 2:** Univariate and multivariate analysis of infection in ICU patients with severe heatstroke (binary logistic regression).

Variable	Univariate		Multivariate	
	OR (95% CI)	P	OR (95% CI)	P
Demographic				
Age (Y)	1.003 (0.949–1.061)	0.906		
Vital signs				
Temperature (°C)	1.642 (1.064–2.533)	0.025		
Heart rate (bpm)	1.048 (1.027–1.069)	< 0.001		
Vasopressors n (%)	22.312 (4.253–117.054)	< 0.001	45.467 (1.460–1416.275)	0.030
Organ				
TBIL (μmol/L)	1.039 (1.018–1.061)	<0.001		
SCR (μmol/L)	1.028 (1.016–1.040)	< 0.001	1.026 (1.005–1.048)	0.017
PLT (10^9^/L)	0.980 (0.970–0.989)	< 0.001		
CK (U/L)	1.000 (1.000–1.000)	0.514		
LYM (10^9^/L)	0.833 (0.476–1.458)	0.522	0.167 (0.049–0.572)	0.004
GCS	0.779 (0.704–0.862)	< 0.001		
GD n (%)	47.500 (13.530–166.755)	< 0.001	14.338 (1.063–193.415)	0.045
Lab result				
WBC (10^9^/L)	0.987 (0.896–1.088)	0.794		
PCT (ng/mL)	1.056 (0.986–1.131)	0.123		
CRP n (%)	0.819 (0.210–3.196)	0.774		
Interventions during admission				
MV n (%)	14.286 (4.258–47.930)	< 0.001		
Score				
SOFA	1.626 (1.346–1.964)	< 0.001		

Abbreviations: TBIL: total bilirubin, SCR: serum creatinine, CK: creatine kinase, PLT: platelet, WBC: white blood cell, LYM: lymphocyte count, PCT: procalcitonin, GCS: Glasgow Coma Scale, SOFA: Sequential Organ Failure Assessment, GD: gastrointestinal dysfunction, CRP: c-reaction protein, MV: mechanical ventilation.

### ROC curve for predicting secondary infection in patients with heatstroke based on the predictive model and independent risk factors

**Fig 5:**
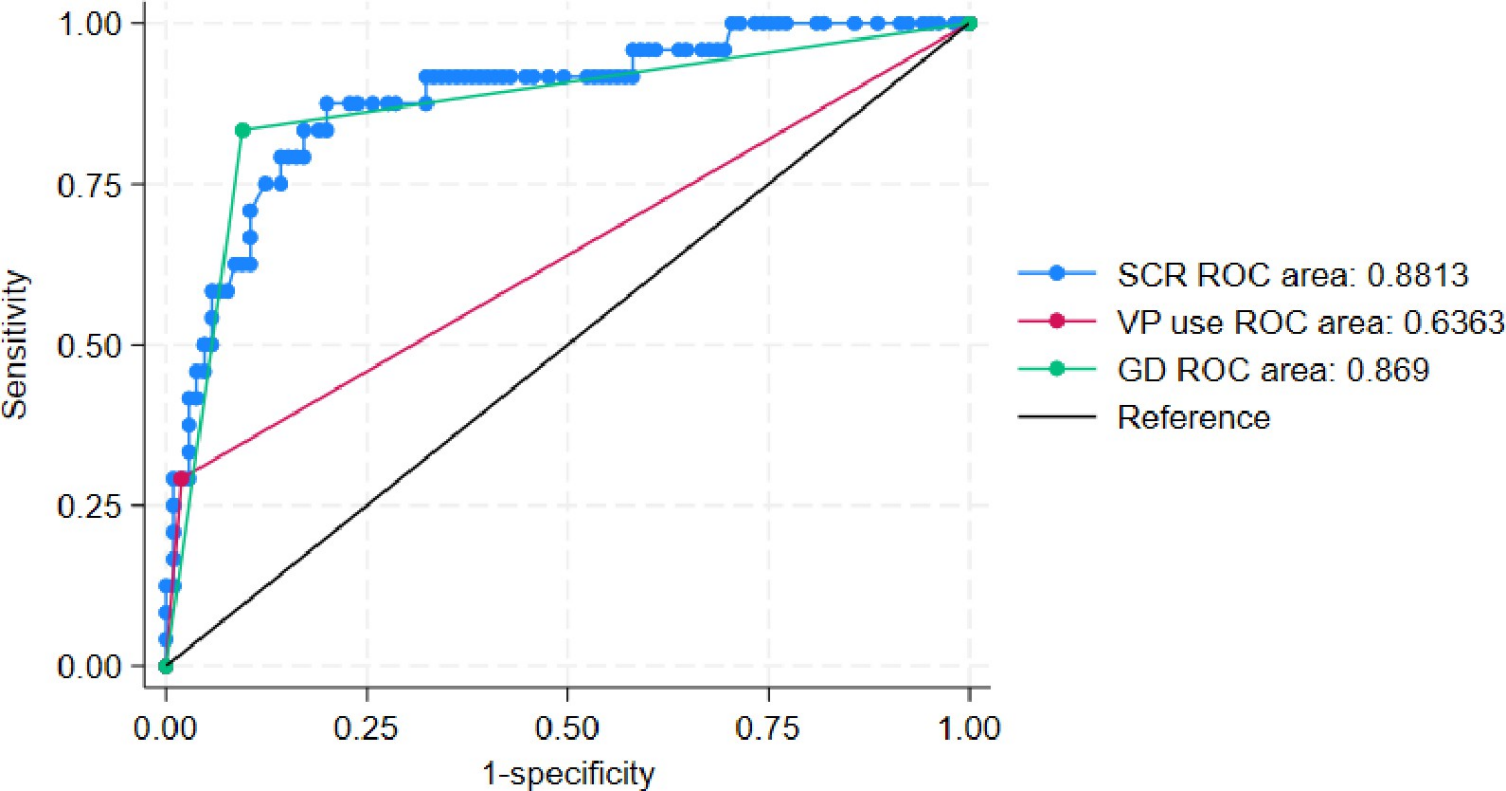
ROC curve for the prediction model, vasopressors use, serum creatinine levels, and gastrointestinal dysfunction in predicting secondary infection in patients with heatstroke.

The ROC curve generated using the predictive equation indicated the predictive performance of vasopressor use, gastrointestinal dysfunction, and serum creatinine levels (Fig 5). The area under the curve (AUC) of serum creatinine for predicting secondary infection in patients with heatstroke was 0.889 (95% CI, 0.812–0.96; P < 0.001), the cut-off value was 136 μmol/L, sensitivity was 87.5%, specificity was 80.8%, and Youden index was 0.683. The AUC of gastrointestinal dysfunction for predicting secondary infection in severe heatstroke was 0.869 (95% CI, 0.776–0.961; P < 0.001), sensitivity was 83.3%, specificity was 90.4%, and Youden index was 0.737. The AUC of vasopressor use for predicting secondary infection in patients with heatstroke was 0.636 (95% CI, 0.498–0.775; needle 0.054), sensitivity was 29.2%, specificity was 98.1%, and the Youden index was 0.683. The AUC of the prediction model incorporating three predictors (vasopressor use, serum creatinine levels, and gastrointestinal dysfunction) for predicting secondary infection in patients with heatstroke was 0.969 (95% CI, 0.935–1.003; P < 0.001), sensitivity was 91.7%, specificity was 93.3%, and Youden index was 0.850.

### Clinical outcomes of heatstroke with infection

Hospital death was considered the major outcome, while length of ICU stay, total length of hospital stay, and duration of mechanical ventilation were considered secondary outcomes (Table 3). Regarding the major outcome, results from multiple factors showed no significant increase in the mortality rates of patients with heatstroke and infections (OR = 1.513; 95% CI, 0.063–36.457, P = 0.799). However, in terms of the outcomes, the mechanical ventilation time (OR = 2.764; 95% CI, 1.735–4.405, P = 0.044) and total length of hospital stay (OR = 1.012; 95% CI, 1.002–1.021, P = 0.027) of patients with heatstroke after infection was significantly prolonged. However, the length of ICU stay did not differ significantly between groups.

**Table 3:** Regression analysis of prognosis prediction of infection in ICU severe heatstroke patients.

Outcome	Univariate		Multivariate	
	OR (95% CI)	P	OR (95% CI)	P
Mortality	51.500 (10.275–258.139)	< 0.001	1.513 (0.063–36.457)	0.799
MV LOS, day	2.786 (1.756–4.421)	< 0.001	2.764 (1.735–4.405)	0.044
ICU LOS, day	1.047 (1.010–1.087)	0.013	1.017 (0.977–1.059)	0.600
HOS LOS, day	1.013 (1.001–1.025)	0.028	1.012 (1.002–1.021)	0.027

Abbreviations: MV LOS: length of mechanical ventilation, ICU LOS: length of ICU stay, HOS LOS: total length of hospital stay.

### Constructing a nomogram based on the prediction model

**Fig 6:**
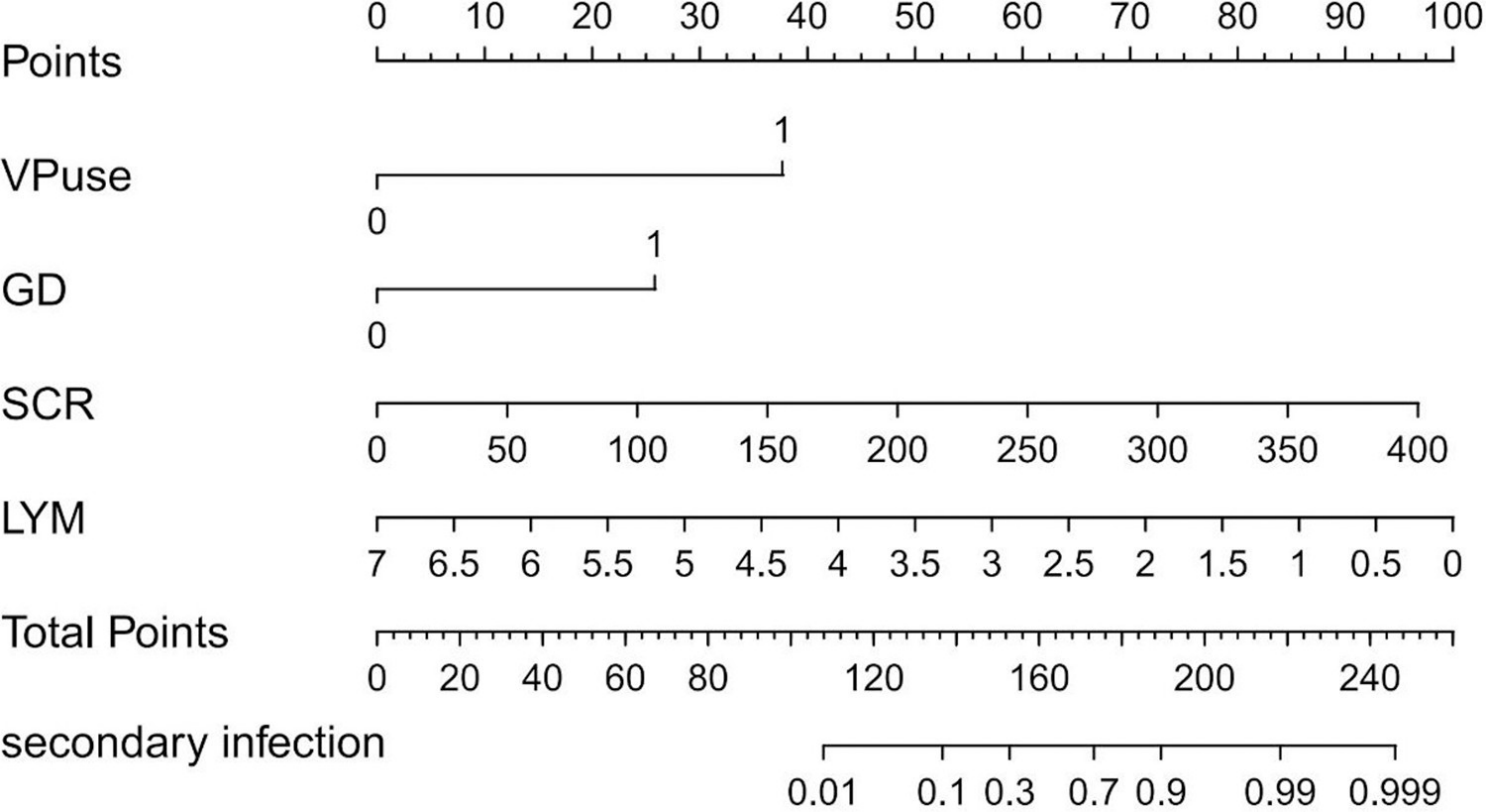
Nomogram for predicting secondary infection in patients with heatstroke.

**Fig 7:**
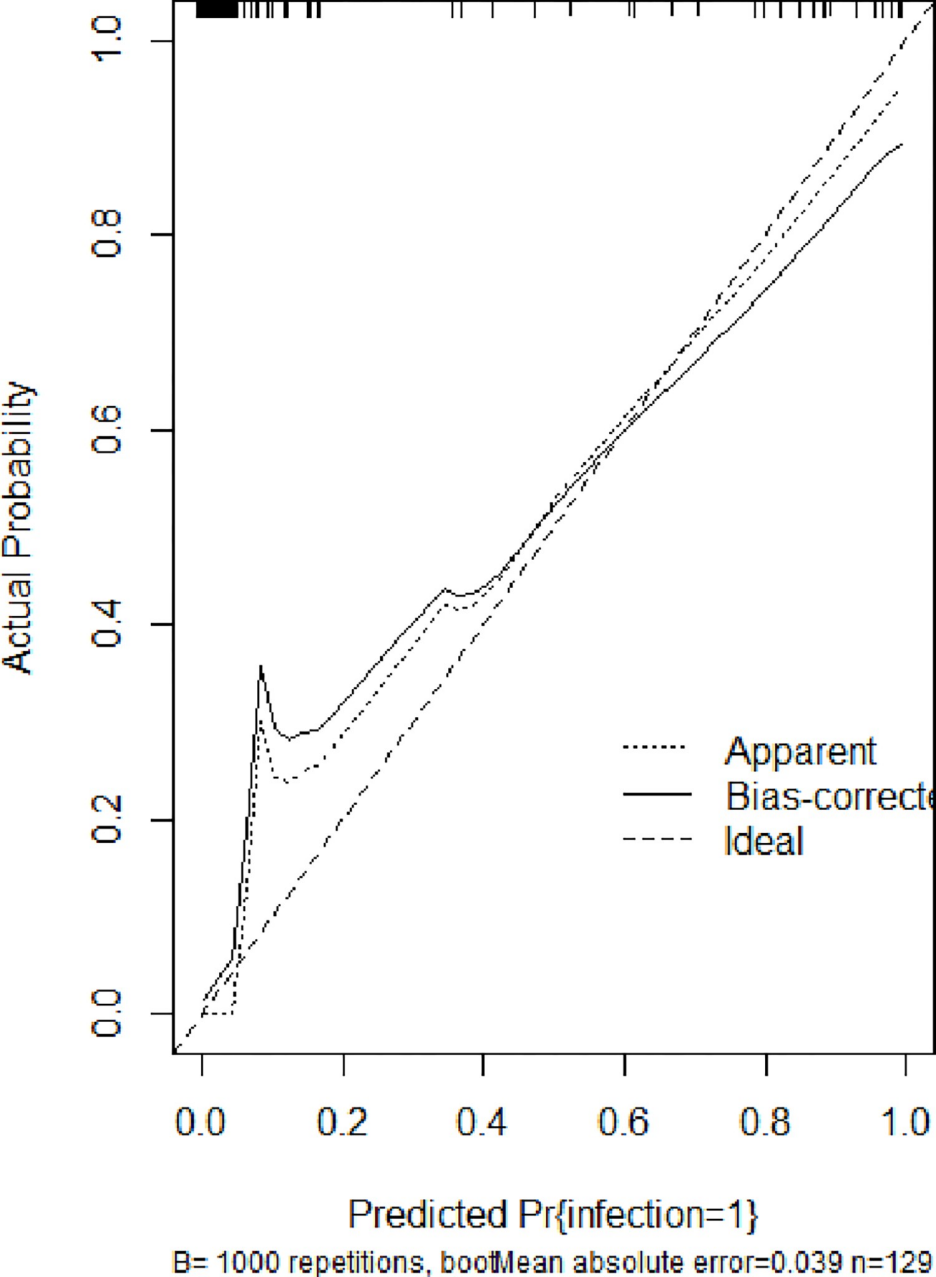
Calibration curve of the nomogram for predicting secondary infection in patients with heatstroke.

A nomogram (Fig 6) was constructed according to vasopressor use, serum creatinine levels, gastrointestinal dysfunction, and lymphocyte count. The bootstrap repeated sampling method (1000 times) was used to verify the calibration of the training and verification queues of the model (Fig 7). The model had good calibration (B = 1000, error = 0.039, n = 129), and the ideal curve fit well.

### Accuracy evaluation of prediction model nomogram

**Fig 8:**
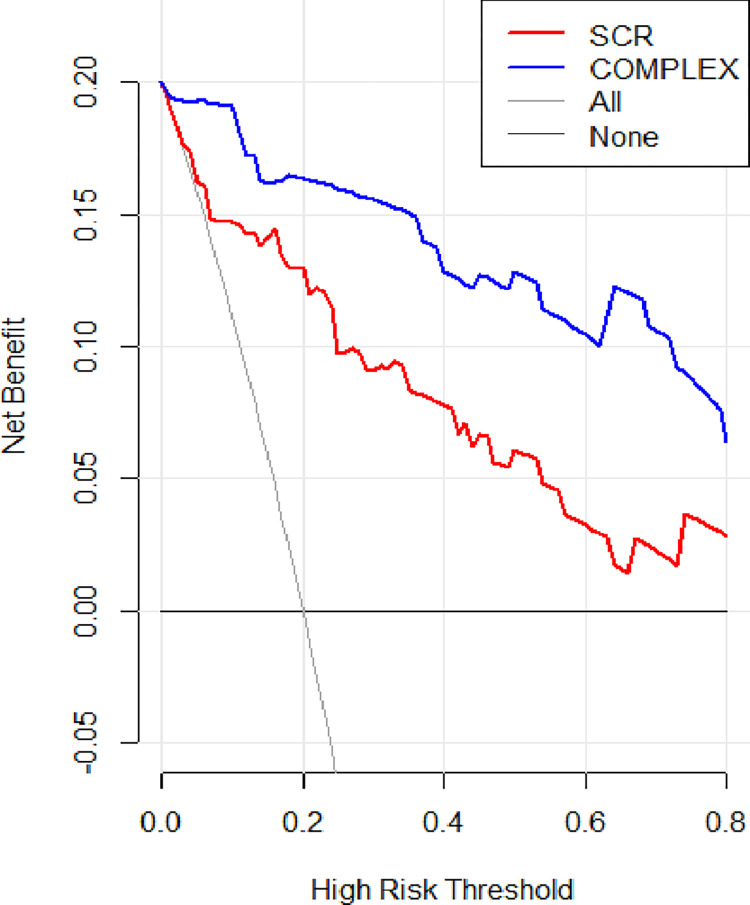
Decision curve analysis (DCA) comparison of the prediction model and serum creatinine.

**Fig 9:**
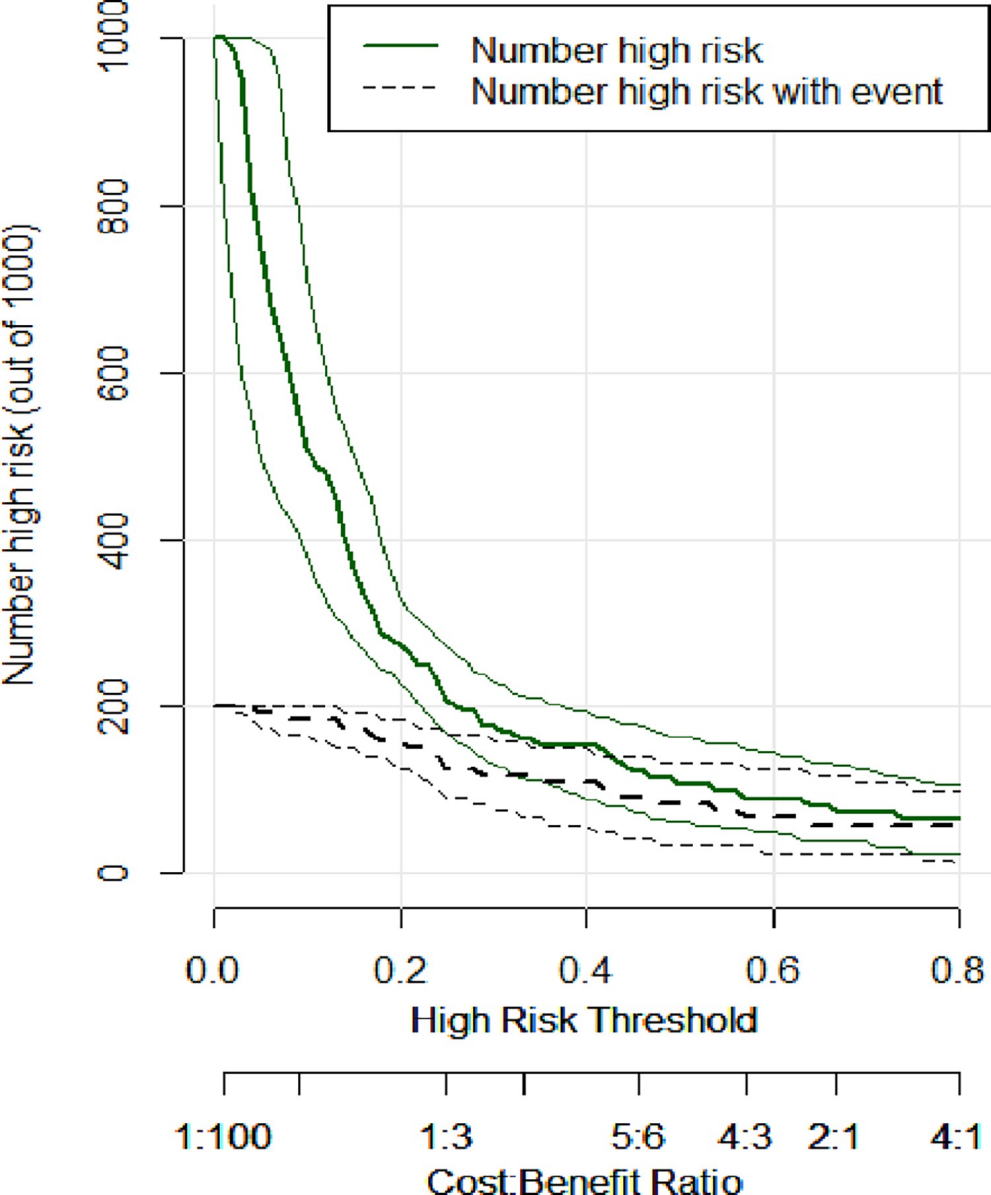
Clinical impact curve (CIC) of the serum creatinine.

**Fig 10:**
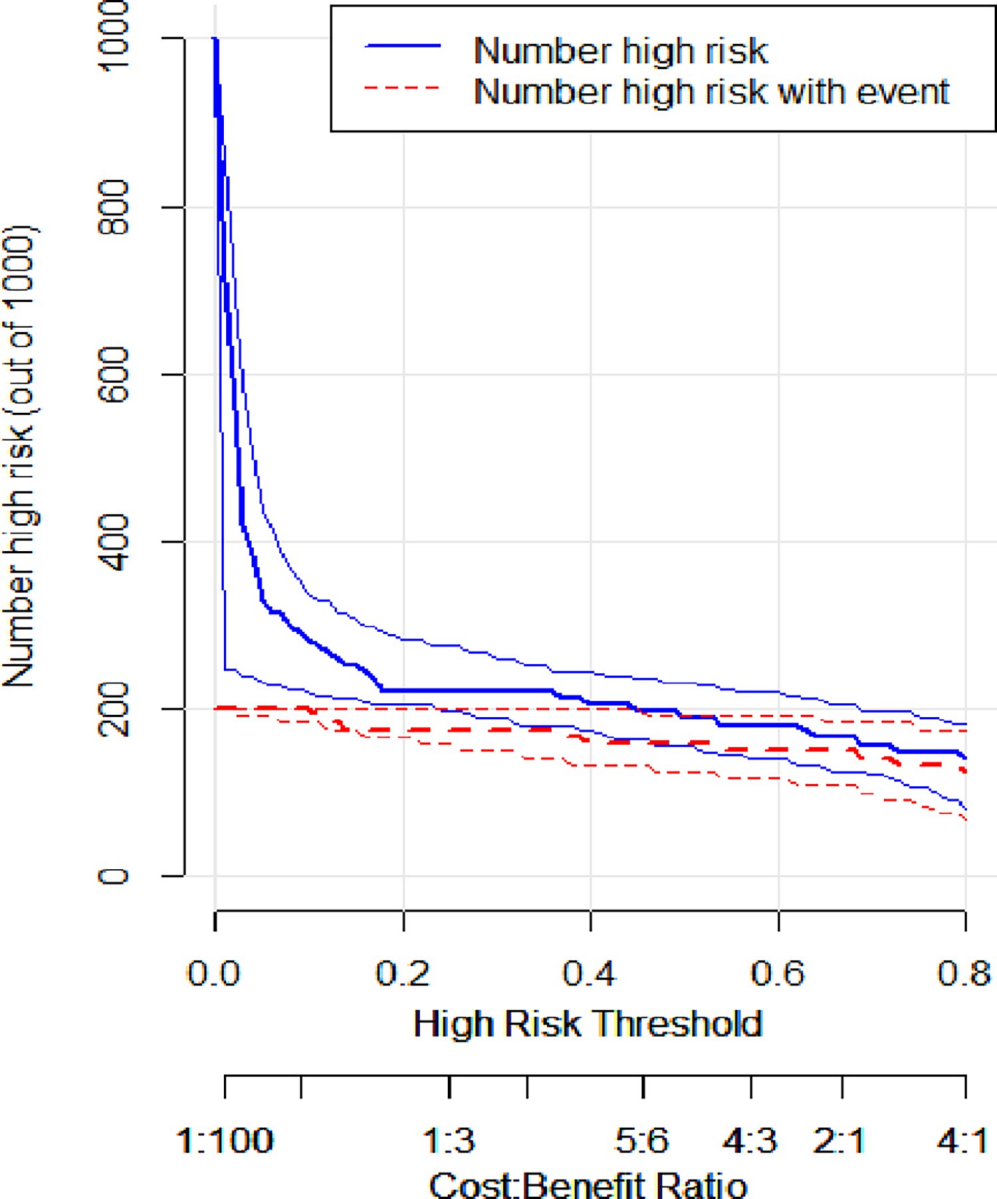
Clinical impact curve (CIC) of the prediction model.

As the ROC curve of serum creatinine and the prediction model intersect within a certain area, the difference in the predictive efficacy of the two could not be accurately described. Hence, decision curve analysis (DCA) was performed, and a clinical impact curve (CIC) was constructed to evaluate the efficacy of the prediction model in clinical decision-making. The clinical net benefit of the prediction model surpassed the “treat-all” and “treat-none” strategies within a broad range of threshold probabilities (Fig 8). Moreover, the area was larger than that of serum creatinine, which had high predictive power in the ROC curve, suggesting that the predictive efficiency of the model was stronger. Additionally, the solid line area represented the predicted number of secondary infections, and the dotted line area represented the actual number of secondary infections in the CIC (Figs 9 and 10). If the two regions had a high degree of integration, consistency was deemed strong, indicating a robust consistency between high-risk patients (predicted by the model) and patients who experienced an adverse outcome. Thus, the prediction model had a better net clinical benefit and clinical effect than serum creatinine.

## Discussion

Vasopressors, gastrointestinal dysfunction, and elevated serum creatinine levels at admission were independent risk factors for secondary infection in patients with heatstroke, while an increased lymphocyte count was an independent protective factor. Based on these variables, a nomogram of the prediction model was constructed; the test efficiency of the calculation model was superior. Heatstroke patients with infections experienced significantly prolonged mechanical ventilation and hospital stays.

Norepinephrine plays an important role in immunosuppression among patients with sepsis [10, 11]. Although no evidence links norepinephrine to aggravating infections [12], using vasopressors may induce certain immunosuppressive effects. For example, norepinephrine has been associated with moderate immunosuppression and promotion of bacterial growth, potentially increasing the risk of infection [10]. Similarly, the current study reveals that the use of vasoactive drugs, primarily norepinephrine, is a risk factor for secondary infections in heatstroke. While using vasoactive drugs as a risk factor in severe illnesses might be associated with disease severity, in this study, with a SOFA score > 0.05, it may not correlate with severity but rather be intrinsic to the vasoactive drugs. Some patients experience hypovolemic shock and require vasopressors for stabilization upon admission, which may increase the risk of infection.

The incidence of gastrointestinal dysfunction was also significantly higher in the infection group than in the non-infection group, and symptoms of severe diarrhea and gastrointestinal bleeding were observed upon admission. Gastrointestinal dysfunction is an important aspect of heatstroke pathophysiology, often beginning with intestinal leakage [13–15]. Others proposed a model of heatstroke endotoxemia [16], showing that exposure to high temperatures and exercise stress can cause gram-negative bacteria to translocate from the gut to the intravascular system, promoting lipopolysaccharide (LPS) transfer from these bacteria to the bloodstream [17, 18]. Moreover, blood LPS concentrations can reach thresholds that trigger systemic inflammatory response syndrome, a major driver of coagulation disorders and multiorgan failure in sepsis [19, 20]. Lim et al. proposed a Dual Pathway Model for heatstroke pathogenesis [20], revealing that heatstroke is triggered by two independent and sequentially activated pathways. In addition to the immediate toxic effects of heat, endotoxemia and systemic inflammation, known as the “hot sepsis” pathway [17], can induce changes in intestinal permeability and promote LPS transport into the circulatory system. Under LPS stimulation, glycolytic and oxidative metabolism pathways in patients with sepsis are significantly downregulated, resulting in “immune paralysis” [21] and increased susceptibility to infections [22]. Thus, heatstroke and gastrointestinal dysfunction may trigger the entry of LPS into the bloodstream, leading to immune paralysis and increased risk of infection.

When body temperature exceeds the normal range by 2°C, the glomerular filtration rate begins to decline, kidney function deteriorates progressively [23], and serum creatinine and urea nitrogen levels gradually increase. The current study identified kidney injury as a risk factor for secondary infections after heatstroke. The pathological mechanism underlying this relationship may include fluid overload, hyperinflammatory states, and immunosuppression associated with renal replacement therapy and indwelling deep venous catheters [24]. Oliguria, fluid retention, tissue edema, and gastrointestinal mucosal edema may occur after acute kidney injury, promoting intestinal bacterial translocation and increasing the risk of latent sepsis or abdominal infection [25]. In addition, increased pulmonary vascular permeability due to fluid overload can aggravate pulmonary edema or promote ventilator-associated lung injury [26], increasing the risk of lung infection [27]. Impaired kidney function, metabolism, or clearance leads to dysregulation of pro- and anti-inflammatory responses [28]. Acute kidney injury is characterized by a high inflammatory state, reduced cytokine clearance, and elevated levels of systemic cytokines, such as interleukin (IL)-17A, IL-6, IL-8, IL-1β, IL-12, and tumor necrosis factor (TNF)-α, which aggravate lung injury and increase the risk of lung infection [29, 30]. Simultaneously, this highly inflammatory state inhibits the immune system function [31] and is not conducive to infection clearance [32]. In addition, uremic toxins and abnormal metabolites secondary to acute renal injury in the inflammatory state weaken the inflammatory response, reduce the bactericidal effect [33], lead to immunosuppression, and promote infection [34, 35]. High concentrations of uremic toxins and pro-inflammatory cytokines, such as resistin, suppress immune responses [36]. Acute kidney injury leads to urea accumulation, which when converted into ammonia in the intestine, disrupts the tight junctions of the intestinal epithelium, increasing the occurrence of bacterial translocation and the risk of intestinal flora imbalance [37], intestinal inflammation, and intestinal leakage, contributing to immunosuppression and subsequent infection [38]. In addition, patients receiving kidney replacement therapy for acute kidney damage are at a higher risk of nosocomial infections [39].

Lymphocytes form the foundation of the adaptive immune system and response, with an important role in evaluating the immune status of patients with sepsis [40, 41]. Immunosuppression is a recognized cause of increased susceptibility to secondary infection and patient mortality [42]. Persistent T and B lymphocytopenia underpins immunosuppression in sepsis [43] and may be a powerful indicator of immunosuppression in critically ill patients [44]. Lymphocytopenia on ICU admission and its persistence at day 3 have also been associated with an increased risk of ICU-acquired infections [45]; persistent lymphocytopenia can also predict increased mortality at 28 days. The current multifactorial analysis revealed that patients with heatstroke after secondary infection had significantly lower lymphocyte counts than those without infection. Hence, heatstroke may induce immune deficiency, resulting in decreased lymphocyte counts, ultimately increasing the risk of infection.

Furthermore, disease severity was higher in the secondary infection group. After adjusting for disease severity, secondary infection did not significantly increase mortality in patients with heatstroke; however, it did extend the total length of hospital stay. Most studies have shown that ICU-acquired infections are independent risk factors for in-hospital mortality [46], even after adjusting for APACHE II or SOFA scores and age. Fagon et al. suggested that ventilator-associated pneumonia, the most common ICU-acquired infection, has a mortality rate of up to 50%, with at least 25% of deaths directly attributable to secondary infection rather than to the underlying disease [47]. However, ICU-acquired infections may not affect long-term patient survival [48]. A prospective study in the Netherlands showed that patients with sepsis and ICU-acquired infections were more severely ill at admission; however, secondary infections in the ICU contributed minimally to overall mortality [49]. Combined with the current results, the extent of early organ damage may be more likely to cause death than infection.

The dominant view is that invasive surgery is a high-risk factor for infection. However, in the current study, all patients in the infection group had deep vein catheters and urinary catheter indentations at admission. Univariate analysis of the risk factors for secondary infection revealed high values for the two variables with extremely poor fitting of the multifactor regression model; thus, the two variables were unsuitable for multifactorial analysis. Consequently, the clinical effects of these two variables on secondary infections could not be definitively ruled out. Moreover, others have suggested that secondary infections may not be associated with invasive procedures but with disease severity. A prospective study [50] suggested that ICU-acquired infections may be more closely tied to the severity of the patient’s underlying conditions than invasive procedures.

Finally, this study integrated multiple influencing factors of secondary infection in heatstroke to construct a nomogram model to more accurately predict high-risk patients with severe heatstroke and secondary infection. The calibration curve of the model suggested that it fitted well with the ideal curve (B = 1000, error = 0.039, n = 129) and had good predictive accuracy. DCA results indicated that the nomogram model had a high clinical utility value. The CIC visually showed that the nomogram had a superior overall net benefit within the threshold probabilities, indicating that the model possessed significant predictive value. In conclusion, the nomogram model had high predictive and clinical application values, providing a reference basis for clinicians to identify high-risk patients with severe heatstroke secondary infection at an early stage, prevent infection, and reduce the risk of death.

## Limitations

First, this single-center retrospective study included only 129 qualified cases; the sample size was small. Second, the study spanned an extended period, during which the early clinical diagnostic capabilities for detecting secondary infections were relatively limited, and the detection rate of pathogens was low. Therefore, the sample size of the infection group was relatively small. Third, due to the limitations of clinical testing technology, we could not collect a detection index of immune cell activity. In addition, the lymphocyte count was only collected within the first 24 h of ICU admission without tracking the longitudinal trends during patients’ hospitalization in the ICU. Therefore, this approach has certain limitations in evaluating the early immune status of patients with heatstroke. Finally, all qualified cases included in this study were young males who had experienced exertional heatstroke. Therefore, the generalizability of the findings to other age groups or classical heatstroke cases may be limited.

## Conclusion

The use of vasopressors, increased serum creatinine levels, and gastrointestinal dysfunction are independent risk factors for secondary infection in patients with heatstroke, while an increased lymphocyte count is an independent protective factor. After adjusting for age and severity of illness in patients with heatstroke, the duration of mechanical ventilation and total length of hospital stay of patients with severe heatstroke and secondary infections were prolonged. The nomogram model constructed by integrating influencing factors has good predictive value and clinical utility, providing a basis for clinicians to accurately identify patients with secondary infections in severe heat stroke at an early stage. However, this model requires further validation in future studies.

## Supporting information

S1 Dataset (XLSX)
